# Role of iRhom2 in intestinal ischemia-reperfusion-mediated acute lung injury

**DOI:** 10.1038/s41598-018-22218-8

**Published:** 2018-02-28

**Authors:** Jee Hyun Kim, Jihye Kim, Jaeyoung Chun, Changhyun Lee, Jong Pil Im, Joo Sung Kim

**Affiliations:** 1 Department of Internal Medicine, Seoul National University Boramae Hospital, Seoul National University College of Medicine, Seoul, Republic of Korea; 2 Department of Internal Medicine and Liver Research Institute, Seoul National University College of Medicine, Seoul, Republic of Korea; 3 Department of Internal Medicine and Healthcare Research Institute, Healthcare System Gangnam Center, Seoul National University Hospital, Seoul, Republic of Korea

## Abstract

Intestinal ischemia-reperfusion (I/R) may cause acute systemic and lung inflammation. However, the detailed mechanism of this inflammatory cascade has not been fully elucidated. Inactive rhomboid protein 2 (iRhom2) is essential for the maturation of TNF-α converting enzyme (TACE), which is required for TNF-α secretion. We evaluated the role of iRhom2 in a mouse model of intestinal I/R using iRhom2 knockout (KO) and wild-type (WT) mice. Lung injury following intestinal I/R was significantly attenuated in iRhom2 KO mice compared with WT mice. After intestinal I/R, lungs from iRhom2 KO mice showed significantly lower myeloperoxidase (MPO) activity and markedly reduced cell apoptosis associated with a decreased level of active caspase 3 and decreased TUNEL staining compared with lungs from WT mice. TNF-α levels were elevated in the serum and lungs of WT mice with intestinal I/R and significantly reduced in iRhom2 KO mice with intestinal I/R. iRhom2 may play a critical role in the pathogenesis of acute lung injury (ALI) after intestinal I/R and thus may be a novel therapeutic target for ALI after intestinal I/R injury.

## Introduction

Intestinal ischemia-reperfusion (I/R) injury develops when the blood flow to the intestines decreases, followed by the reestablishment of the blood supply to the ischemic tissue. Intestinal I/R injury results in intestinal mucosal barrier dysfunction, which may cause severe local and systemic inflammation and subsequent distant organ injury. This injury is potentially serious and can be life threatening; however, the mortality rates in patients with acute intestinal I/R are constant and range from 60–80%^1–3^. Therefore, a new treatment strategy for intestinal I/R is needed.

Acute lung injury (ALI), a medical condition characterized by widespread inflammation in the lung with an acute onset, is the most serious complication of intestinal I/R injury^4,5^. Although several pathophysiologic mechanisms of ALI in intestinal I/R have been suggested, the detailed molecular mechanism is not fully understood. Inflammatory mediators during intestinal I/R include reactive oxygen species, platelet-activating factor, chemokines and cytokines^6–10^. Among these mediators, TNF-α is the most widely studied. High levels of TNF-α are associated with an inflammatory response that leads to many diseases, such as ALI and other chronic lung diseases^11,12^. Animal treatments with anti-TNF-α antibodies and experiments in TNF-α receptor 1-deficient animals have revealed a central role for TNF-α in mediating tissue injury and systemic inflammation in intestinal I/R^9^.

Soluble TNF-α is cleaved from membrane-bound TNF-α by TNF-α converting enzyme (TACE, ADAM17)^13–15^. TACE is an essential enzyme responsible for TNF-α release and is required for the cleavage of other ligands, including epidermal growth factor receptor (EGFR) ligands^16^. Inactive rhomboid protein 2 (iRhom2) was recently identified as an essential regulator of TACE maturation in immune cells^17^. iRhom2 is predominantly expressed in immune cells, particularly in macrophages, and its expression is significantly up-regulated in response to lipopolysaccharide (LPS) stimulation^18,19^. In iRhom2-deficient macrophages, LPS-induced release of TNF-α is significantly inhibited by the failure of TACE maturation. Thus, iRhom2 may be an attractive novel therapeutic target for TNF-α-dependent inflammatory diseases.

The aim of this study was to evaluate the role of iRhom2 in the development of ALI resulting from intestinal I/R.

## Materials and Methods

### Cell culture

The murine macrophage cell-line, RAW 264.7 (Korean Cell Line Bank 40071, Seoul, Korea), was cultured, as described previously^20^.

### Mice

C57BL/6 mice (wild-type, WT) were purchased from Orient (Seongnam, Korea), and iRhom2 knockout (KO) mice, on a C57BL/6 background, were obtained from Dr Tak W. Mak (University of Toronto, Toronto, Canada)^18^. iRhom2 gene KO was confirmed in iRhom2 KO mice by PCR genotyping of colonic tissue DNA (Supplementary Figure 1). Male mice were maintained under specific pathogen-free (SPF) conditions in the Center for Animal Resource and Development of Seoul National University (Seoul, Korea). The mice were supplied a standard chow until they reached the desired age (7–8 weeks) and body weight (20–24 g). The mice were weighed weekly starting at an age of 3 weeks to compare body weight between the two types of mice.

### siRNA-mediated knockdown of iRhom2

To clarify whether iRhom2 regulates TNF-α secretion in macrophages, RAW264.7 cells were transfected with iRhom2 small interfering RNA (siRNA) before LPS stimulation, and TNF-α secretion by LPS-stimulated RAW264.7 cells was measured by enzyme-linked immunosorbent assay (ELISA). Before LPS stimulation, RAW264.7 cells were transfected with iRhom2 or control siRNA using Lipofectamine RNAiMax (Invitrogen)^17,18,20^. Twenty-four hours after transfection, the cells were stimulated with LPS (1 µg/ml LPS for 4 h), and the concentration of TNF-α in the culture supernatants was measured using a commercially available ELISA kit (R&D Systems, Minneapolis, MN, USA).

### Intestinal ischemia-reperfusion model

Intestinal I/R was performed as previously reported^21^. iRhom2 KO and WT mice were randomized into an intestinal I/R group (each n = 10) or a sham laparotomy group (each n = 5). The mice in the intestinal I/R group underwent 1 h of ischemia followed by 3 h of reperfusion. Intestinal I/R was induced by the complete clamping of the superior mesenteric artery (SMA) with a microvascular clip. After 1 h of occlusion, intestinal perfusion was reestablished by removing the clip. Sham-operated mice underwent identical surgical interventions and time courses without SMA clamping. We performed 1 h of intestinal ischemia followed by 3 h reperfusion because a previous pilot experiment showed that this time course provoked the most severe pulmonary inflammation in the WT mice without significant mortality.

### Intestinal and lung histological examinations

Histological changes in the intestine and lung were evaluated. Tissue samples, including small intestine and lung tissues, were fixed in a buffered 10% formalin solution, paraffinized and stained with hematoxylin and eosin (H&E). All histopathological assessments were performed in a blinded fashion by a pathologist who was unaware of the study details. The small intestine and lung injuries were graded in accordance with previously described scoring systems^22^.

### Myeloperoxidase activity

Myeloperoxidase (MPO) activity, which reflects neutrophil infiltration, in the lung tissues was measured as described previously^22^.

### Caspase 3 activity

Immunohistochemistry (IHC) staining for active caspase 3, a marker of apoptosis, was measured in the lung tissues as previously described^23^. The immunoreactivity of caspase 3 was assessed by determining the percentage of positive cells using a visual scoring system and was classified as 0 (no staining), 1+ (<10%), 2+ (10–30%), 3+ (31–60%) and 4+ (61–100%), as previously reported^24^.

### TUNEL assay

The terminal deoxynucleotidyl transferase dUTP nick end labeling (TUNEL) assay was performed in the lung tissues as previously described^25^. The TUNEL assay detects DNA fragmentation that results from apoptosis. The apoptotic index was calculated based on the ratio of TUNEL-reactive cells to the total number of cells in each of three randomly selected, nonadjacent fields (magnification: x 200)^26^.

### TNF-α and IL-6 in the blood and lungs

The levels of TNF-α and IL-6 in the serum and lung homogenates were measured using a commercially available ELISA kit according to the manufacturer’s instructions.

### Statistical analysis

All data are expressed as the mean ± standard deviation (SD) and were analyzed using GraphPad Prism software version 5.0 (GraphPad, La Jolla, CA). Data were analyzed using a two-way Student’s t-test or one-way analysis of variance (ANOVA). *P* < 0.05 was considered statistically significant.

### Ethical considerations

This study protocol was approved by the Institutional Animal Care and Use Committee of Seoul National University (IACUC No. SNU-160612). The experimental procedures were performed in accordance with the Guide for the Care and Use of Laboratory Animals, published by the National Institutes of Health.

## Results

### iRhom2 siRNA inhibits TNF-α secretion in LPS-stimulated RAW264.7 macrophages

**Figure 1:**
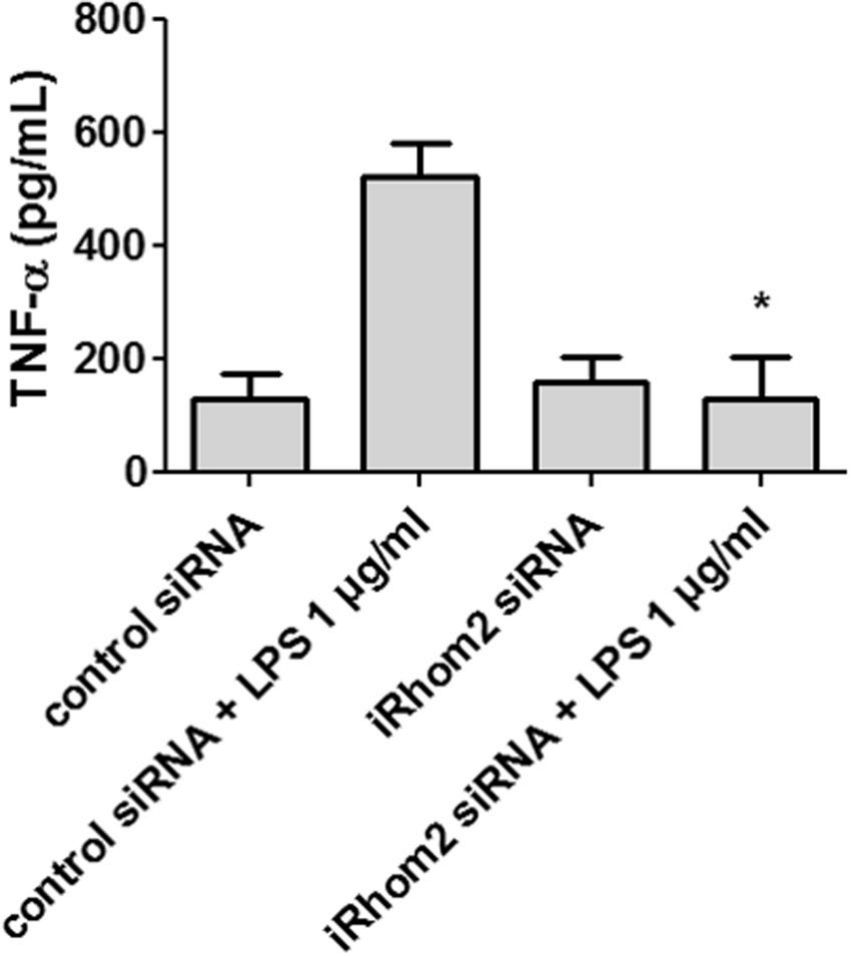
siRNA-mediated iRhom2 knockdown inhibits TNF-α secretion in LPS-stimulated macrophages. RAW264.7 cells were transfected with iRhom2 or control siRNA before LPS stimulation, and TNF-α secretion by LPS-stimulated RAW264.7 cells was measured by ELISA. TNF-α secretion by LPS-stimulated macrophages was significantly inhibited by iRhom2 knockdown. *P < 0.05 versus the control siRNA + LPS group.

The effect of iRhom2 siRNA treatment on LPS-induced TNF-α secretion by macrophages was examined by ELISA. The results indicated that stimulating RAW264.7 macrophages with LPS led to TNF-α secretion. This effect was significantly decreased by siRNA-mediated iRhom2 knockdown (Fig. 1), establishing iRhom2 as a key molecule in the release of TNF-α from macrophages.

### iRhom2 deficiency alleviates lung injury after intestinal I/R

**Figure 2:**
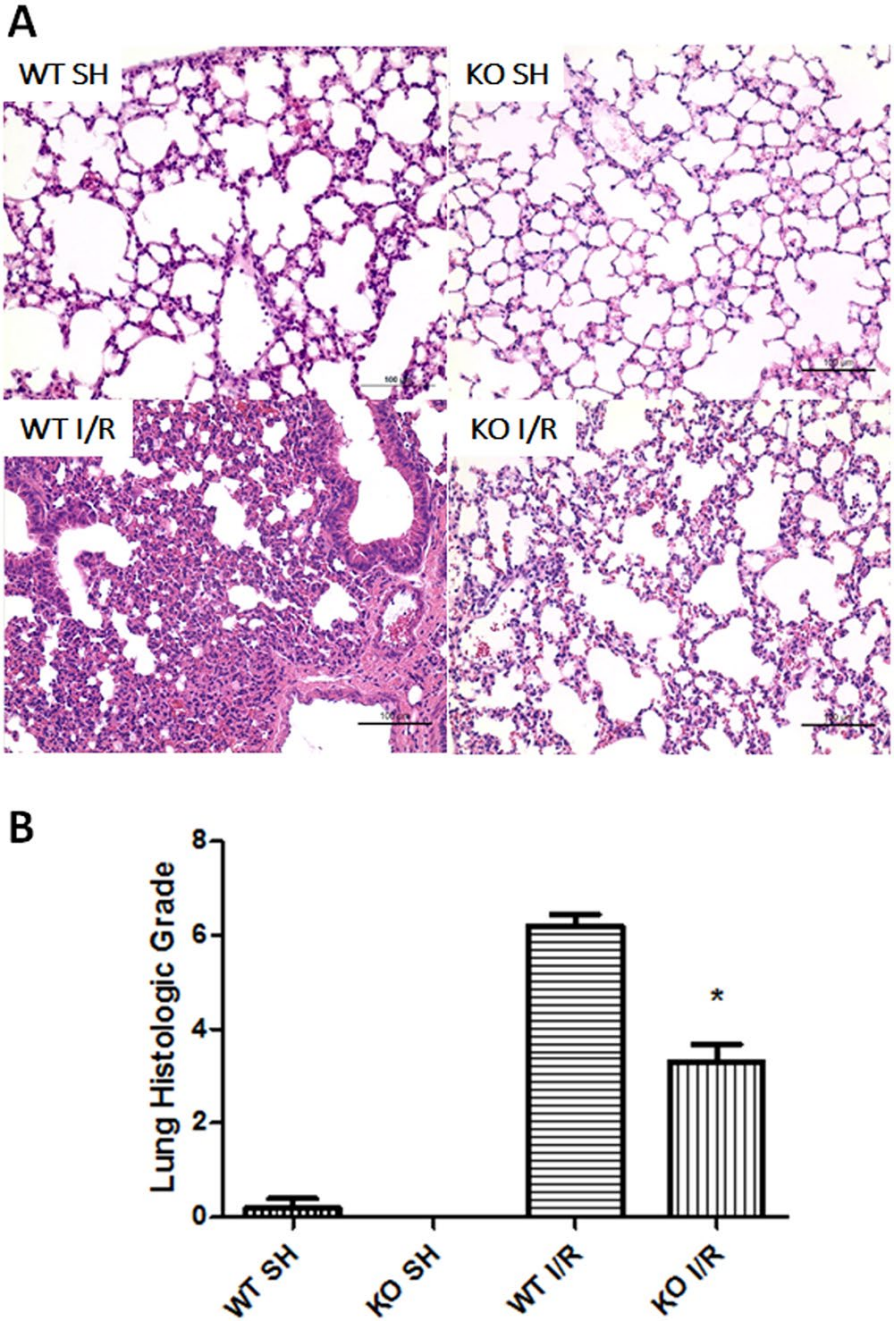
iRhom2 deficiency ameliorates lung injury after intestinal ischemia-reperfusion (I/R). (**A**) Representative histological sections of hematoxylin and eosin (H&E)-stained lung tissues from wild-type (WT) mice and iRhom2 knockout (KO) mice are shown (x200). Scale bars: 100 μm. The iRhom2 KO and WT mice were randomized into an intestinal I/R group (each *n* = 10) and a sham laparotomy group (each *n* = 5) as follows: KO SH (sham-operated KO group), WT SH (sham-operated WT group), KO I/R (KO with intestinal I/R group), and WT I/R (WT with intestinal I/R group). Lung injury following intestinal I/R was significantly attenuated in the iRhom2 KO mice compared with the WT mice. (**B**) Lung injury was assessed by using a modified scoring system with three criteria: alveolar edema/exudates, hemorrhage and interstitial/alveolar cellular infiltration. *P < 0.05 versus the WT I/R group.

After 1 h of ischemia followed by 3 h of reperfusion, the WT mice showed severe lung damage, as indicated by substantial alveolar edema, inflammatory cellular sequestration and hemorrhage, whereas the sham-operated group exhibited normal lung histology (Fig. 2A). However, these changes were significantly alleviated in the iRhom2 KO mice. Statistical analysis of the histologic scores showed that iRhom2 deficiency significantly attenuated the severity of lung injury following intestinal I/R (Fig. 2B).

### iRhom2 deficiency inhibits lung neutrophil infiltration after intestinal I/R

**Figure 3:**
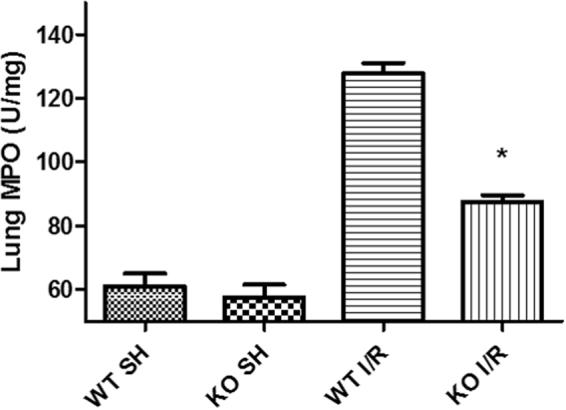
iRhom2 deficiency alleviates lung neutrophil infiltration after intestinal ischemia-reperfusion (I/R). The myeloperoxidase (MPO) activity in the lung tissues was measured to quantitate the accumulation of neutrophils in the lung. The lung MPO activity was significantly decreased in the iRhom2 knockout (KO) mice compared with the wild-type (WT) mice after intestinal I/R. *P < 0.05 versus the WT I/R group.

Compared with the sham control group, neutrophil infiltration, as measured by MPO activity, increased in the lungs of WT mice subjected to intestinal I/R (Fig. 3). The MPO activity was significantly lower in the iRhom2 KO mice subjected to intestinal I/R than in the WT mice.

### iRhom2 deficiency reduces lung cell apoptosis after intestinal I/R

**Figure 4:**
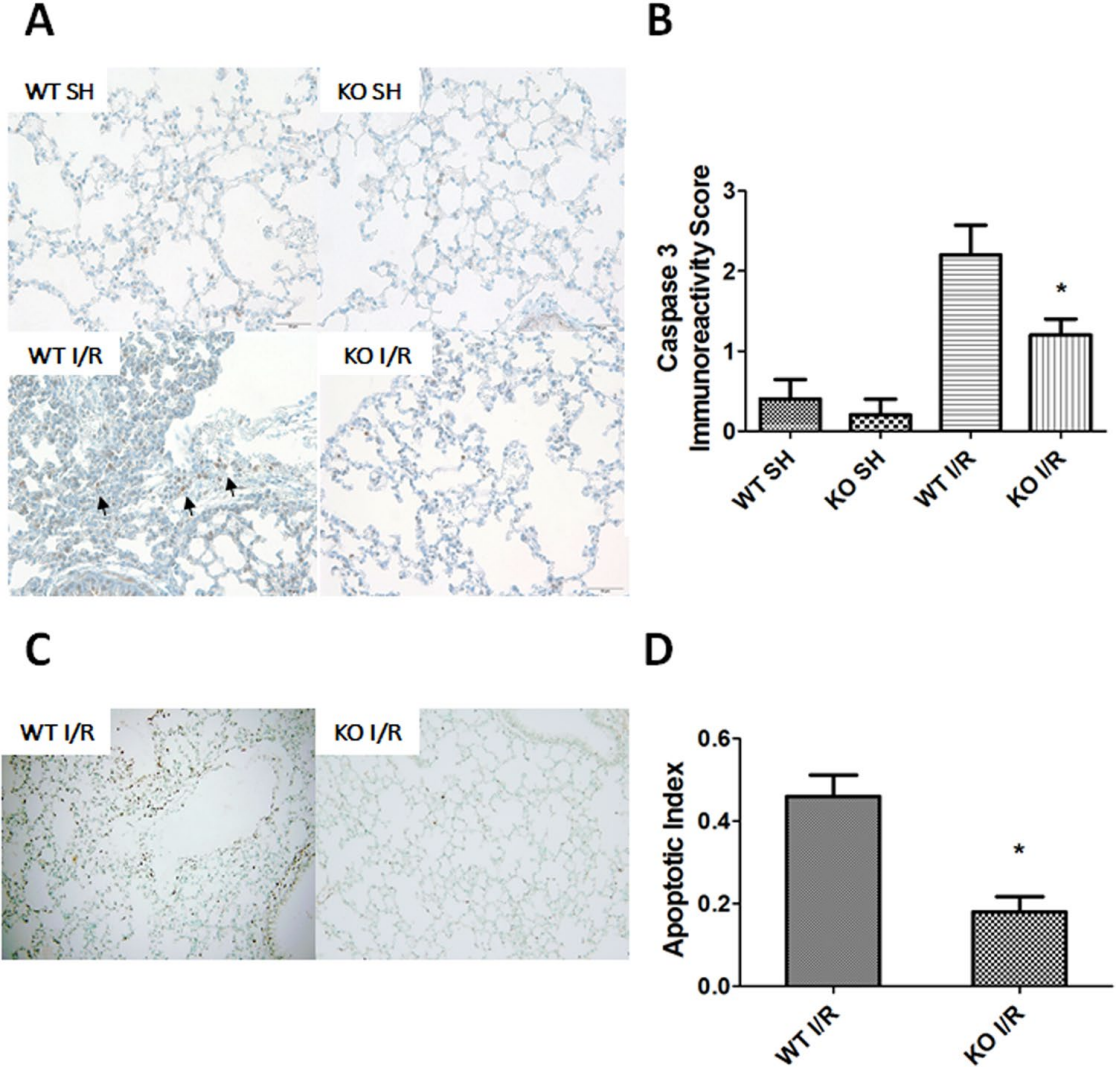
iRhom2 deficiency reduces lung cell apoptosis after intestinal ischemia-reperfusion (I/R). (**A**) Representative images of active caspase 3 immunohistochemical staining in lung tissues (cleaved caspase 3 [diluted 1:1000]) are shown (x400). Scale bars: 50 μm. After intestinal I/R injury, the active caspase 3 protein levels were substantially reduced in the iRhom2 knockout (KO) mice compared with the wild-type (WT) mice. The arrows indicate active caspase 3-positive cells. (**B**) The immunoreactivity score of caspase 3 in the lung tissues was significantly decreased in the iRhom2 KO mice compared with the WT mice. The immunoreactivity of caspase 3 was assessed by determining the percentage of positive cells using a visual scoring system and was classified as 0 (no staining), 1+ (<10%), 2+ (10–30%), 3+ (31–60%) and 4+ (61–100%). *P < 0.05 versus the WT I/R group. (**C**) Terminal deoxynucleotidyl transferase dUTP nick end labeling (TUNEL) assays of the lungs after intestinal I/R in the WT and iRhom2 KO mice are shown (x200). TUNEL staining was reduced in the iRhom2 KO mice compared with the WT mice after intestinal I/R. The black-brown coloring indicates positive staining, and green indicates the contrast background staining. (**D**) The apoptotic index was also markedly reduced in the lungs of iRhom2 KO mice. The apoptotic index was calculated based on the ratio of TUNEL-reactive cells to the total number of cells in each of three randomly selected, nonadjacent fields. *P < 0.05 versus the WT I/R group.

IHC staining for active caspase 3 and a TUNEL assay were subsequently performed to examine apoptosis in the lungs (Fig. 4). In the WT mice, active caspase 3 expression was virtually absent in the sham-operated group and was dramatically increased in pulmonary and inflammatory cells after intestinal I/R (Fig. 4A). However, this change was markedly attenuated in the iRhom2 KO mice. The immunoreactivity score of caspase 3 in the lung tissues of iRhom2 KO mice was significantly lower than that in the WT mice (Fig. 4B). In addition, TUNEL staining showed only a few positive cells in the iRhom2 KO mice, whereas many positive cells that were characterized as inflammatory cells or alveolar epithelial and endothelial cells were observed in the WT mice after intestinal I/R (Fig. 4C). The apoptotic index was also markedly reduced in the lungs of iRhom2 KO mice (Fig. 4D).

### iRhom2 deficiency does not affect intestinal injury after intestinal I/R

**Figure 5:**
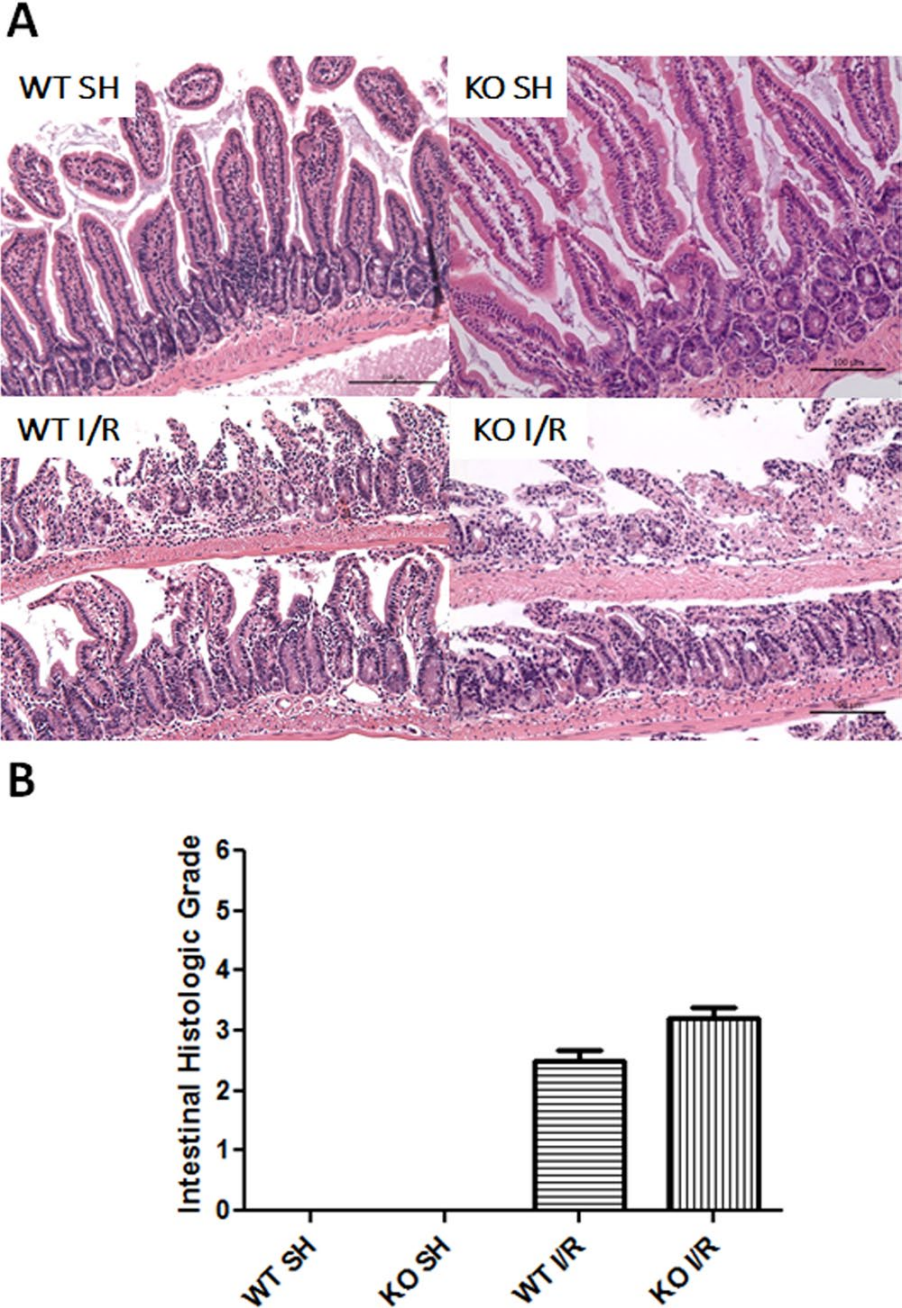
iRhom2 deficiency does not affect intestinal injury after intestinal ischemia-reperfusion (I/R). (**A**) Representative histological sections of H&E-stained intestines from the wild-type (WT) and iRhom2 knockout (KO) mice are shown (x100). Scale bars: 100 μm. The intestinal injury was not different between the iRhom2 WT and KO mice. (**B**) The severity of the intestinal injury was assessed using a histological grading system based on the following criteria (score: 0–5): preservation of the villi, epithelial degeneration/necrosis, erosion or ulceration, and mucin depletion.

Reperfusion after SMA occlusion in both the iRhom2 KO and WT mice caused intestinal injury, characterized by epithelial erosion, inflammatory cell infiltrates and destruction of the villi (Fig. 5). The intestinal injury severity was assessed using a histological grading system based on the following criteria (score: 0–5): preservation of the villi, epithelial degeneration/necrosis, erosion or ulceration, and mucin depletion. The intestinal injury severity was not significantly different between the iRhom2 WT and KO mice.

### Alterations in inflammatory cytokines after intestinal I/R

**Figure 6:**
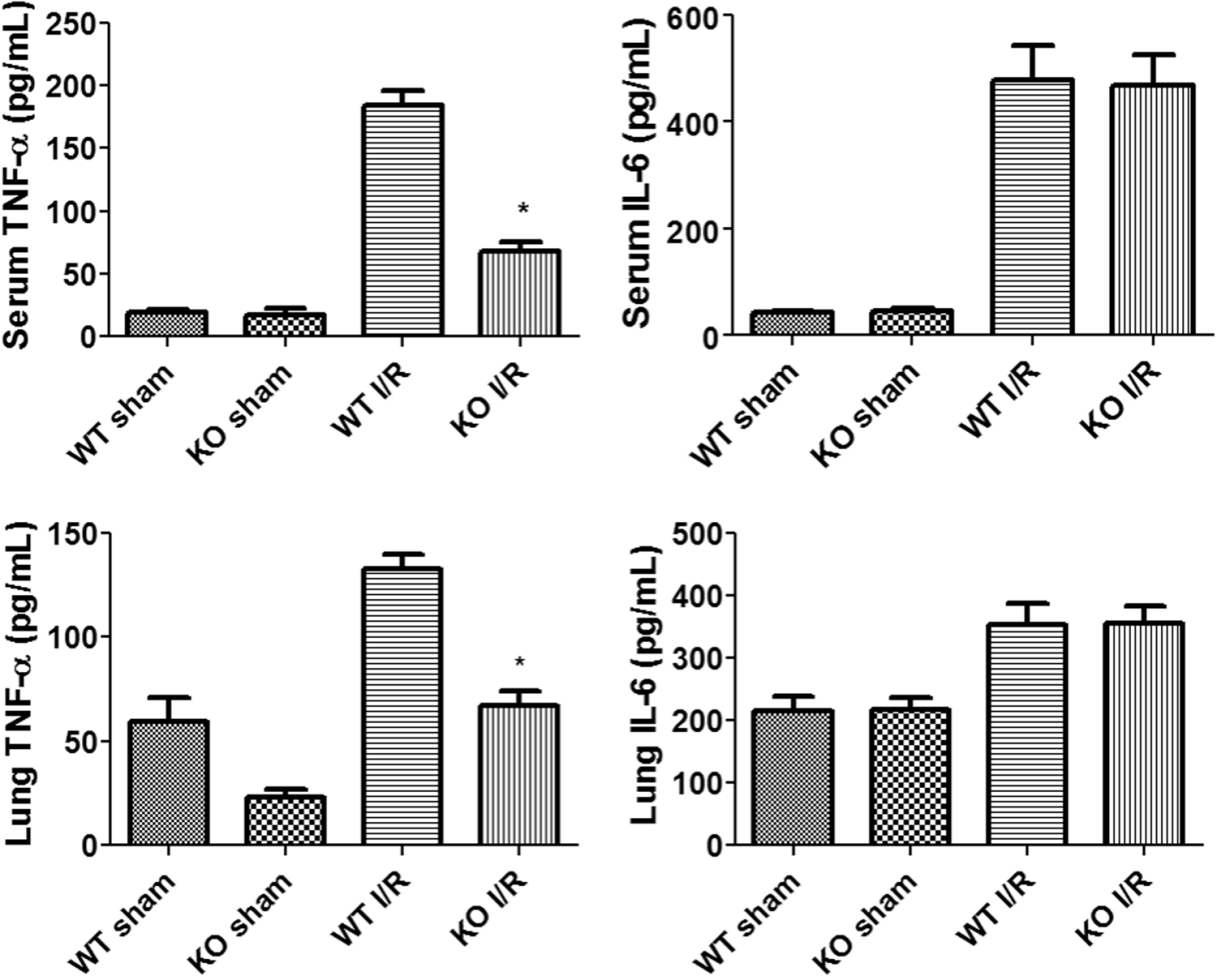
Alterations to inflammatory cytokines after intestinal ischemia-reperfusion (I/R). Inflammatory cytokine proteins (TNF-α and IL-6) in the serum and lung were measured by ELISA. *P < 0.05 versus the WT I/R group.

We measured the levels of two inflammatory cytokines, TNF-α and IL-6, in the serum and lung homogenates (Fig. 6). In the WT mice, the levels of TNF-α and IL-6 in the serum and lungs were significantly up-regulated by intestinal I/R injury. The iRhom2 KO mice subjected to intestinal I/R also showed a similar increase in the IL-6 levels in the serum and lungs compared with the WT mice. However, in the iRhom2 KO mice, the I/R-induced release of TNF-α was markedly reduced in the serum and lungs.

## Discussion

In the present study, we confirmed our hypothesis that iRhom2 played pivotal roles in the remote lung inflammation induced by intestinal I/R injury. Lung injury following intestinal I/R was significantly attenuated in the iRhom2 KO mice compared with the WT mice. iRhom2 deficiency reduced neutrophil activity and cell apoptosis in the lung. Following intestinal I/R, the TNF-α levels increased in the serum and lungs of WT mice. However, TNF-α production during intestinal I/R was markedly decreased in the iRhom2 KO mice. Unlike lung injury, the intestinal injury severity was not different between the iRhom2 WT and KO mice. To the best of our knowledge, the present study is the first to evaluate the role of iRhom2 in intestinal I/R.

The intestinal I/R injury procedure performed in this study is a simple and reproducible model of local and systemic inflammation with significant effects on lung and gut homeostasis^27–29^. We performed 1 h of intestinal ischemia followed by 3 h of intestinal reperfusion, and this condition was regarded as a model of injury with inflammation characterized by neutrophil infiltration into the intestine and lung that peaked after 3 h without significant mortality. Similarly, in a previous study, 45 min of intestinal ischemia followed by 4 h of intestinal reperfusion was used to induce a mild injury in the lungs without mortality^21^.

In mice, intestinal I/R injury is followed by severe local intestinal and remote lung tissue pathology, characterized by a marked neutrophil influx, edema formation, hemorrhage, and tissue destruction^8,9,30,31^. In addition to tissue damage, marked systemic inflammation is observed, as assessed by increased serum levels of inflammatory cytokines and chemokines^31^. Similarly, in the present study, intestinal I/R injury induced intestinal and lung injury and up-regulated the expression of inflammatory cytokines such as TNF-α and IL-6.

TNF-α is involved in the pathogenesis of various inflammatory diseases. Since TACE was first discovered to be essential for TNF-α release, TACE blockade has been considered as a treatment for TNF-α-mediated inflammatory diseases. However, blockade of TACE inhibits not only TNF-α secretion but also EGFR signaling. TACE KO mice are not viable and show a phenotype similar to that of mice lacking EGFR signaling^32^. Furthermore, mice with markedly reduced TACE activity show increased susceptibility to acute colitis, probably due to a lack of EGFR signaling. However, since iRhom2 is a myeloid-specific regulator of TACE maturation, iRhom2 blockade might be an effective way to treat inflammatory conditions without inhibiting EGFR signaling. Accordingly, in the present study and in previous studies, iRhom2 KO mice were viable and appeared normal, without morphological defects or growth retardation (Supplementary Figure 2)^18,33,34^.

In the present study, the iRhom2 KO mice showed attenuated lung injury and lower levels of TNF-α in the serum and lungs compared with the WT mice. Consistent with previous studies, in RAW264.7 macrophages, TNF-α secretion was increased by LPS stimulation and significantly decreased by siRNA-mediated iRhom2 knockdown^17,18^. A previous study showed that other inflammatory cytokines, such as IL-1β, IL-6 and IL-12, were induced normally in iRhom2-deficient macrophages^18^. Taken together, these results could suggest that iRhom2 and the subsequent release of TNF-α play an important role in mediating systemic inflammation after intestinal I/R. Several studies have established a role for TNF-α in mediating both local and systemic inflammation during I/R injury^11,35–37^. TNF-α induces the expression of adhesion molecules on endothelial cells and the recruitment of inflammatory cells into the lung, and an anti-TNF-α antibody reduces the severity of lung injury^37^. TACE inhibition reduces the release of soluble TNF-α and the severity of LPS-induced lung injury^11^. However, the role of TNF-α in intestinal I/R-mediated ALI has been controversial. A previous report showed that neither the neutralization of soluble TNF-α with antibody treatment nor the lack of TNF-α or its receptors diminished lung inflammation upon intestinal I/R^21^.

Neutrophils are known to play a critical role in the pathogenesis of ALI. TNF-α stimulates neutrophils in several disease models^38–40^. Recombinant human TNF-α stimulates a neutrophil respiratory burst and lysozyme release and significantly elevates the neutrophil respiratory response^41^. In this study, the influx of neutrophils into the lungs in the iRhom2 KO mice was reduced compared with that in the WT mice. Cell apoptosis has been proposed as another possible mechanism in ALI^42,43^. Previous studies have reported that TNF-α induces caspase activation in endothelial cells, which results in the induction of apoptosis and endothelial cell dysfunction^44,45^. iRhom2 KO mice with ALI exhibited a significantly lower apoptosis intensity than the WT mice, and our findings suggested that iRhom2 deficiency directly and/or indirectly resulted in less cell apoptosis in the lungs. TNF-α has also been shown to induce necroptosis, a non-apoptotic form of cell death, in different cell types^46^. The precise cell death mechanism involved in intestinal I/R-induced ALI could be characterized in future studies.

Interestingly, there was no significant difference in the severity of the local intestinal injury between the iRhom2 WT and KO mice. This result may be explained by the function of transmembrane TNF-α in intestinal inflammation. Although the role of transmembrane TNF-α is not fully understood, data suggest that both soluble TNF-α and transmembrane TNF-α are important in the inflammatory response, particularly in local inflammation. Transmembrane TNF-α transmits signals as both a ligand and a receptor in TNF-α-producing cells^47^. Recently, several studies have reported that transmembrane TNF-α plays a key role in local inflammation^48–50^. In transgenic mice expressing a mutant transmembrane TNF-α, transmembrane TNF-α is sufficient to induce localized tissue injury and chronic inflammatory arthritis^51,52^. Moreover, a recent study in a mouse model of T cell-mediated colitis reported that only neutralization of transmembrane TNF-α induces remission of experimental colitis^53^. In iRhom2-deficient macrophages, TNF-α biogenesis is normal, but TACE proteolytic activity is defective. Therefore, transmembrane TNF-α in the iRhom2 KO mice may cause local intestinal inflammation similar to that of the WT mice.

In conclusion, the iRhom2 protein may be associated with the pathogenesis of intestinal I/R. iRhom2 deficiency has a protective role in the systemic inflammation induced by intestinal I/R. Although the detailed molecular mechanisms of iRhom2 and subsequent TNF-α signaling in intestinal I/R remain to be clarified, our data suggest that iRhom2 may be a novel therapeutic target for ALI after intestinal I/R injury.

## Electronic supplementary material

Supplementary Information
