# Author Correction: Role of iRhom2 in intestinal ischemia-reperfusion-mediated acute lung injury

**DOI:** 10.1038/s41598-018-25425-5

**Published:** 2018-05-01

**Authors:** Jee Hyun Kim, Jihye Kim, Jaeyoung Chun, Changhyun Lee, Jong Pil Im, Joo Sung Kim

**Affiliations:** 1 Department of Internal Medicine, Seoul National University Boramae Hospital, Seoul National University College of Medicine, Seoul, Republic of Korea; 2 Department of Internal Medicine and Liver Research Institute, Seoul National University College of Medicine, Seoul, Republic of Korea; 3 Department of Internal Medicine and Healthcare Research Institute, Healthcare System Gangnam Center, Seoul National University Hospital, Seoul, Republic of Korea

Correction to: *Scientific Reports* 10.1038/s41598-018-22218-8, published online 28 February 2018

The Acknowledgements section in this Article is incomplete.

“This work was supported by a National Research Foundation of Korea (NRF) grant by the Korean government (The Ministry of Science and ICT; NRF-2015R1A2A2A04002733).”

should read:

“This work was supported by a National Research Foundation of Korea (NRF) grant by the Korean government (The Ministry of Science and ICT; NRF-2015R1A2A2A04002733) and grant No 04-2016-0540 from the SNUH Research Fund.”

