# Supplementary material for: Role of iRhom2 in intestinal ischemia-reperfusion-mediated acute lung injury
Source: Sci Rep. 2018 Feb 28;8:3797. doi: 10.1038/s41598-018-22218-8 (PMC5830505; doi:10.1038/s41598-018-22218-8)
Supplement: Supplementary file 1 — Supplementary Information [file 41598_2018_22218_MOESM1_ESM.pdf]

Role of iRhom2 in intestinal ischemia-reperfusion-mediated acute lung injury

Jee Hyun Kim[1], Jihye Kim[2], Jaeyoung Chun[2], Changhyun Lee[3], Jong Pil Im[2] and Joo Sung Kim[2,3]

Supplementary Info File

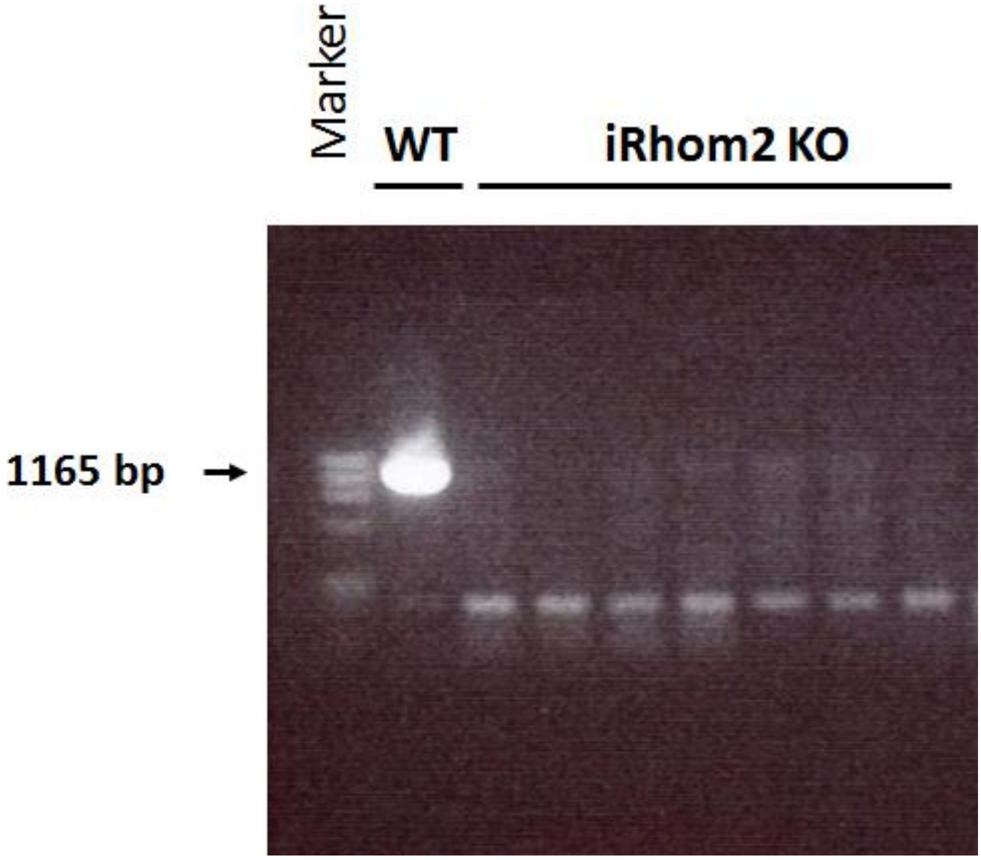

Supplementary Figure 1. PCR genotyping of iRhom2 knockout (KO) mice. iRhom2 gene KO was confirmed in the iRhom2 KO mice by PCR genotyping of colonic tissue DNA. The arrow indicates the amplified iRhom2 fragment (1165 bp).

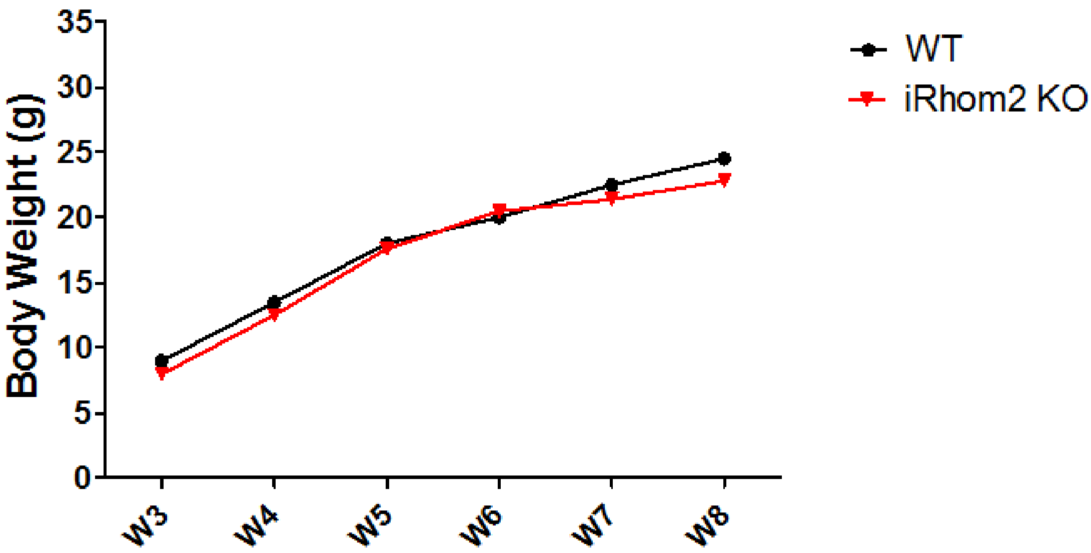

Supplementary Figure 2. Body weight in wild-type (WT) and iRhom2 knockout (KO) mice. The WT and iRhom2 KO mice were weighed weekly starting at an age of 3 weeks. No significant difference was observed between the iRhom2 KO mice and WT mice at any time point.
